# "The Hospital" Nursing Mirror

**Published:** 1898-11-19

**Authors:** 


### "The Hospital" Nursing Mirror.

Being the Nursing Section of "The Hospital."

[Contributions for this Section of "The Hospital" should be addressed to the Editor, The Hospital, 28 & 29, Southampton Street, Strand, London, W.C., and should have the word "Nursing" plainly written in left-hand top corner of the envelope.]

### News from the Nursing World.

### AN ACQUISITION AT VICTORIA PARK HOSPITAL.

The nurses at the Hospital for Diseases of the Chest at Victoria Park are most fortunate in having had recently placed at their service a sitting-room which is really a drawing-room. In former times this was the matron's linen-room, and it is a large, lofty apartment, with rafters of dark oak. The walls are tinted green, with a deep red dado, the floor has a stained margin, and is covered with a handsome Oriental carpet, a bargain because of its size. The furniture is in good taste, there being a number of easy chairs, couches, occasional and writing tables, and a piano by Broadwood; the pictures are good, they and the cushions are the gift of the matron. Altogether the new acquisition is an unqualified success, and must increase the attractiveness of the hospital to nurses as a residence. There is a well stocked bookcase from which the nurses may take books and as they please, the only formality being to enter the name of any book taken from the room.

### NEW NURSES' HOME AT ORMSKIRK.

On the 12th inst. the Countess of Derby opened a new block of buildings, adjoining the Cottage Hospital at Ormskirk, which has just been erected to provide accommodation for the out-patient and administrative departments of the hospital and dispensary, and also for the residence of five district nurses. The Countess was accompanied by Lady Isobel Stanley, the Hon. Arthur Stanley (M.P. for the Division), Lord Skelmersdale, and Lady Bertha Wilbraham. The Rev. Canon Blundell, chairman of the committee, presided, and the formal proceedings took place in the boardroom, where a large and influential gathering was assembled. The new block has cost about £3,000, which is almost defrayed; and an endowment fund, in connection with the work of the district nurses, is proposed in memory of the late Lady Lathom, who was deeply interested in it.

### FOR THE "QUEEN'S" NURSES.

On the 10th inst. the artistes in connection with the Music Halls' Fund gave a matinée at the Empire Music Hall in aid of Queen Victoria's Jubilee Nurses' Institute, from which it is expected that £350 will accrue to the benefit of the institution. Mr. H. J. Hitchins arranged an excellent programme, and there was a crowded audience. All the performers gave their services, and some notable members of "the profession" sold programmes, and thus helped to increase the sum total of the receipts.

### A RED CROSS NURSE'S DEATH.

We regret to chronicle the death of Miss Sylvia Coffin, who bore an important *rôle* in the Red Cross Nursing Service during the late Cuban War. After organising the nursing at Tampa, Miss Coffin accompanied the troops on the transport "Lampasas" to Santiago, and hence to Ponce. Here the troops were landed, and about one hundred sick men took their place. The food was deficient and bad, the water polluted, the vessel filthy, medical and nursing staff weak, and the supplies inadequate. It is not a matter of surprise, therefore, that typhoid in a malignant form appeared, or that, unfortunately, Miss Coffin, in helping to nurse the sick, contracted the disease, and, after a two months' illness, she died at the Post Graduate Hospital, New York.

### PROJECTED NEW NURSES' HOME.

A site within the grounds of the institution has been chosen by the managers of the Northern Infirmary at Inverness for the erection of a new nurses' home. The cost of the building is estimated at £1,471. It will be arranged on two floors, and Messrs. Ross and Macbeth are the architects.

### NIGHT NURSING BY DISTRICT NURSES.

We were asked some little time ago what arrangements were usual with regard to night nursing by district nurses, and, the subject being one of considerable importance, we have made inquiries about it. Miss Peter, the Inspector of the Queen's Nurses, says that they do not, as a rule, undertake night duty unless in special cases. It is unusual in rural districts for the nurse to have the care of more than one serious case at a time, and, when that case is chronic, a good, trustworthy woman can be got to sit up if no friend is available, and the nurse occasionally takes a night to give the friends a rest. The lady superintendents of two large associations inform us that it is quite impossible for them to undertake night nursing without largely increasing their staffs, and that an outside nurse is engaged when necessary for this purpose.

### BEDFORDSHIRE COUNTY NURSES.

The Duchess of Bedford took the chair at the first annual meeting of the Bedfordshire Rural Nursing Association, which was held in the Shire Hall, Bedford, last week. There is much that is satisfactory in the year's work, but much remains to be done, and unconsciously the chief speakers touched upon one of the gravest objections to training an inferior grade of nurse from a fully-trained nurse's point of view. Thanks principally to the grants of the Technical Instruction Committee of the County Council a number of suitable candidates have been sent to London and trained for six months. By this means a probationer obtains the certificate of the L.O.S. and a certificate in general nursing from the institution wherein she was trained, and the Bedfordshire Association guarantees her a salary of 12s. a week and uniform for three years, after which she is free to work where she will, and to charge what she likes. The last words are significant. So much has been written and said upon the controversy of fully-trained versus partially-trained nurses that it seems a fair and pertinent question to ask the advocates of the cottage nursing systems what safeguard they propose to protect the public from mistaking the untrained for the fully-qualified woman in the future. If at the end of three years—the minimum recognised term of training—the partially-trained nurse can flourish a couple of certificates in the faces of an ignorant public, and charge the full price of competent work (if she can get it), the cottage associations are fraught with grave dangers both to the trained nurse and the public. They become little more than cheap manufactories of unqualified nurses, and as such will flood the market to the detriment of those who are fully qualified. The saving in cost between a fully-trained nurse and a half-trained one is nominal, if the cost of training be added to the three years' stipend, and to the expense and worry of finding suitable candidates. We congratulate the association upon the appointment of a paid secretary. It is an admission of the principle that one must pay for what one gets, and very rightly so, too.

### DISTRICT NURSING AT PONTEFRACT.

THE first annual report of the Pontefract District Nursing Association states that "the work has been carried on most satisfactorily, and evidence is forthcoming from all sides that a real and much felt want has been met by the ministrations of the nurse." The committee record their appreciation of the manner in which Nurse Thomson has discharged her duties, and of the skill and kindness which have won for her the gratitude of all her patients. Financially also the association is to be congratulated, having a very fair balance in hand after paying all expenses. Altogether the association has well justified its existence in this its first year's work.

### IN AID OF FAKENHAM NURSES' HOME.

RECENTLY a grand bazaar was held in the Corn Hall, Fakenham, at which £176 were realised for the funds of the local nurses' home. There was a large attendance on the first day, the Countess of Leicester opening it. She was accompanied by Lady Mabel Coke. Heavy rain, however, thinned the gathering for the second day's sale, which was inaugurated by Lady Wilton. The hall was prettily decorated with flags and banners, and the stalls presented an attractive appearance with flowers, fruit, Maderia and Swedish baskets, Algerian vases, pincushions, sweetmeats, as well as a variety of fancy articles.

### FORMATION OF A NEW ASSOCIATION.

A NUMBER of ladies and gentlemen met last week at Kimberley, Nottingham, and inaugurated the Kimberley and District Nursing Association for the purpose of providing trained nurses for the neighbourhood. It was resolved that one or two nurses should be engaged, to live in a home at Kimberley under a superintendent, and that in cases of emergency the association should apply for help to the Nottingham and Notts Nursing Federation. The management will be vested in a general committee formed of subscribers of £1 and upwards, and of representatives from the friendly societies and clubs, the members of which shall contribute sixpence apiece annually. The executive committee is to be composed of ladies elected by the general committee.

### PROGRESS AT CORK.

THE new nursing scheme proposed some time ago at the South Infirmary, Cork, has been adopted. Our readers may remember that we gave an account of it at the time it was approved, and there is little to add. The three trustees appointed to the Nursing Committee are Monsignor Maguire, Canon Harley, and Mr. Newsom. Sister Jane has been appointed staff superintendent nurse. The nurses engaged by the late matron, Miss Woodroffe, are to remain on the same terms if they wish it, and the buildings now set apart for the nurses' dormitories will still be retained for their service.

### SHORT ITEMS.

THE staff of the Ipswich Nurses' Home at present consists of a lady superintendent, fourteen private, three district, and ten cottage nurses; there are also in training one district and two cottage probationers. The district staff is not large enough, and more subscribers are needed to enable it to cope efficiently with the needs of the sick poor. It is satisfactory that the part-paying cottage cases have increased from 115 to 150.—The in-patients of the Cancer Hospital, Brompton, were much interested in a lecture recently given by their chaplain, the Rev. F. W. A. Wilkinson, on his holiday tour through Switzerland and the Italian Lakes. The lecture was illustrated by 70 lantern slides, and forms the first of a series of entertainments.—The late Mrs. Mary Slater, of Heywood House, Bore Street, Lichfield, has left £20 a year from the residue of her estate during the lifetime of her brother-in-law and executor, Mr. J. S. Brown, to the Lichfield Nursing Institution and Invalid's Kitchen. At his death the estate will be vested in trustees, who, after obeying certain stipulations, will have power to apply the same to the support and maintenance of this institution.—Lord Roberts has replied through his secretary to a correspondent that the Army Nursing Sisters are chosen irrespective of their religious persuasions, and that, as the nursing service could not be satisfactorily carried on under other conditions, there is no reason to make special representations of the fact that none of the Sisters are Roman Catholics at headquarters.—Lady Yarborough opened the sale of work held recently at Springfield, Bargate, Grimsby, in aid of the funds of the Grimsby and District Nursing Institution. There was a grand concert in the evening for the same purpose. The work is increasing, and the reserve fund has had to be encroached on, hence the special effort to raise money.—Two nurses have recently died of typhoid fever at the Willesden Isolation Hospital. The second, Nurse Jackson, was a promising nurse who had been in the hospital four years.—A sum of £13 was realised at a jumble sale held at the Corn Exchange, Stamford, recently, for the funds of Queen Victoria's Jubilee Institute for Nurses. The Mayoress and several other ladies interested themselves in it.—Funds to the extent of £50 have been subscribed and promised to support a nurse for Cranham and Upminster. The vicar's wife, Mrs. Cooke, and Mrs. Williams, of Hoppea Hall, have had the project at heart for some time past.—At a public banquet lately given to the Mayor of Wednesbury reference was made to the part he had taken in connection with the Nurses' Institute. Not only has the cost of the erection of the home been defrayed during the Mayor's two years of office, but a sum of £700 has been collected towards the Endowment Fund.

### Nursing in Diseases of the Throat, Nose, and Ear.

By MACLEOD YEARSLEY, F.R.C.S.Eng., Assistant Surgeon to the Royal Ear Hospital, Surgeon-in-Charge of the Department for Diseases of the Throat, Nose, and Ear, the Farringdon General Dispensary, Hon. Surgeon for Diseases of the Throat and Ear, the Governesses' Home, &c.

### VI.—OPERATIONS ON THE THROAT.

THE operations on the throat are both numerous and varied, ranging from the application of the galvano-cautery to excision of the larynx. They include one of the most important operations in surgery, viz., tracheotomy, a procedure of such importance that it has been judged wiser to devote a special article to it and its after treatment.

The operations on the pharynx through the mouth are as follows: Galvano-cautery applications, uvulectomy, tonsillectomy, for peritonsillar abscess. Such operations as cleft-palate belong rather to the province of general surgery, and the nurse will probably have already obtained considerable experience thereof. They will not, therefore, be touched upon.

The galvano-cautery is applied to granular pharyngitis for the destruction of the granules and the obliteration of the enlarged blood-vessels. One of the finer points will be used, together with a tongue depressor. Galvano-puncture is used largely in America for the reduction of large tonsils; a fine point is required, but the method is both tedious and somewhat painful. The galvano-cautery is also used for reducing enlarged veins and adenoid hypertrophy at the base of the tongue. A broad point is generally used for these purposes. For the latter condition a curette or snare, or a special form of guillotine may also be used.

Uvulectomy: For the removal of an elongated uvula either special scissors in conjunction with a pair of long bent forceps or a cord or galvano-cautery snare, or one of the numerous special uvulotomes may be used.

Tonsillectomy: For the reduction of enlarged tonsils a simple guarded bistoury with a pair of forceps, or a tonsillotome may be used. Of the latter instrument there are numberless modifications. The best known and most universally used tonsillotome is that of Mackenzie. The wire snare is also used by some practitioners. When the tonsil does not sufficiently project portions are often punched out with one of the various punch forceps invented for that purpose, the best of which is Kreichman's.

Peritonsillar abscesses require opening with an ordinary bistoury or one specially guarded. The old method of partially surrounding the blade of a curved bistoury with strapping is a dirty one, and should the nurse be asked to prepare a knife for the operation she can quickly do so by slipping a piece of drainage tubing over the blade so as to leave the last half-inch (at the point) uncovered. A tongue depressor should also be handed to the surgeon.

All the above small operations may be done under cocain or eucain. Often the tonsils are removed at the same time as post nasal growths under a general anæsthetic. In these small operations, especially in tonsillectomy, the nurse may be asked to steady the patient's head. In doing so she should cover the hair with a towel, and, standing directly behind the patient, rest his head against her chest, firmly steadying it by placing the hands on either side, the wrists resting on or just behind the ears, so as not to prevent the patient from properly opening his mouth. When tonsils are removed she will greatly assist the operator by pressing them inwards. This is done by placing the fingers just behind the angle of the jaw and pressing gently but firmly inwards and slightly upwards. She must have at hand a basin or a porringer into which the patient may expectorate, and some warm, weak solution of potassium permanganate with which he can wash out his mouth. She should prevent him from hawking and violently expectorating.

Passing now to the larynx, one of the most frequent procedures the nurse will witness is the injection of fluids into that organ. Beyond the fact that she should know a laryngeal syringe when asked for one, this proceeding will not concern her. In the treatment of laryngeal tubercle portions of morbid tissue are frequently removed by means of various curettes or cutting forceps. These present many different patterns, with which the nurse will only become conversant by actual ocular experience. Beyond thus mentioning them nothing will be gained by any further allusion to them. As regards the removal of morbid tissue and growths, the nurse should always see that there are bottles prepared for the preservation of specimens. This applies equally to the nose and ear; the bottles should contain such preserving fluids as the surgeon may prefer.

Operations upon the larynx are not of such a nature as to require much assistance on the part of the nurse; even the instruments and their selection would be undertaken by the surgeon himself. In such operations as laryngo-fissure, subhyoid pharyngotomy, laryngectomy, removal of foreign bodies, &c., the surgeon, if he is a wise man, will look after his own instruments and antiseptics. In these operations the nurse's duties will be the same as in any ordinary general surgical procedure.

The intra-laryngeal operations (operations performed through the mouth) again are not likely to call for more service from the nurse than the steadying of the patient's head and the arrangements for the patient's comfort immediately afterwards.

A few words regarding the operation of intubation of the larynx will not be amiss, although many nurses will have already witnessed it in the wards of the general hospital in which they were trained. O'Dwyer's tubes and the instruments for their insertion and extraction should be (and probably are) well known to the reader; if not, she should lose no opportunity of seeing them or the figures of them in an instrument maker's catalogue. For intubation, the child should be firmly wrapped up in a light blanket, the arms being inside, and held sitting upright upon the nurse's lap. It is best to let the child sit well back, so that its back can be held firmly against the nurse's chest, whilst the latter steadies the little head against her shoulder with one arm. If the nurse's other arm be passed round the patient's body and the latter's feet be held fast between her knees and legs, the child should be powerless to move whilst the surgeon is doing his work. The mouth is held open with a gag by an assistant. The introduction of the tube by a skilled hand does not occupy more than 15 seconds. In extracting the tube the patient requires holding firmly in the same way as for its introduction.

Speaking generally, the duties of the nurse in the surgery of the throat are of more importance in after treatment than during the operation.

### Presentations.

ON October 28th the Mayor (Miles Ashworth, Esq.) and Mayoress of Rochdale gave a farewell dinner to Miss Lucy Smith, the Matron of Rochdale Fever Hospital at the Town Hall. After the dinner the Mayor presented Miss Smith with a beautiful gold watch as a farewell gift from the committee and officers. Miss Smith, who has held the matronship for five years received numerous other gifts on her departure.

### Training in the Provinces.

(*Continued from page* 69.)

### NEWCASTLE ROYAL INFIRMARY (270 Beds).

### Terms of Training.

Candidates for training at the Newcastle Royal Infirmary are required to make a personal application, and can be seen by the matron daily from 1.30 to 2 p.m.; the age considered desirable is from 22 to 30. Probationers are admitted after a three months' trial (during which period they must supply their own uniform), and they then enter into a two years' agreement, taking day and night duty alternately as required. For the first year probationers receive no salary, for the second £10 is paid to them, and the matron has power to retain their services, after the two years, at a salary of £20, a certificate being granted at the end of three years. Staff nurses' salaries range from £24 to £30, and head nurses' (or sisters) from £24 to £36, according to the importance of their office and the length of their service. When appointed after the three months' trial, probationers are supplied with uniform, (four print dresses, four caps, six aprons, and six collars), to be renewed at the discretion of the superintendent of nurses. If a nurse or probationer leaves the hospital before the expiration of twelve months from the date of the last issue of her uniform its estimated value is charged to her. Laundry expenses are defrayed by the hospital. Lectures are given to the nurses during their training by members of the medical and surgical staff, and classes, which all probationers are required to attend, are held by the matron. Three years' training is a necessary qualification for promotion to a post as ward sister. Night duty is taken for three months at a time.

### Hours On and Off Duty.

Probationers and assistant nurses are on duty on alternate days from 7 a.m. to 5 p.m., and from 7 a.m. to 9 p.m. Time for meals must be deducted from the hours on duty. On Sunday those nurses whose turn it is to be on duty all day are allowed two hours' leave of absence. Head nurses go on duty in the morning at 8 o'clock; in other respects their hours are the same as those already given. Night nurses are on duty from 9 p.m. to 8 30 a.m.; half an hour is allowed for the midnight meal. They take their recreation from 9.30 to 12 noon.

The annual holiday for the nursing staff is from three to four weeks, arranged as may be found convenient.

### Meals.

All meals for the day nurses are served in the dining-room; the sisters have their early lunch and afternoon tea sent to their own sitting rooms. Allowances of tea, sugar, &c., are served out weekly to each ward for the sisters there on duty; probationers, staff and assistant nurses, have stores served to their dining room. The day nurses breakfast at 6.30 a.m.; dinner, for which 45 minutes are allowed, is at 12 and 1.30; tea at 3.30 and 4 p.m.; supper at 9. Sisters breakfast at 7.45 a.m.; dinner is served at 1.30; tea at 4 to 4.30; and supper at 9.15. The nurses on night duty have dinner before going to their wards, at 8 p.m.; half an hoar is allowed for tea during the night; and they breakfast at 9 a.m., having a light lunch at 12, before going to bed.

---

### NORFOLK AND NORWICH HOSPITAL (220 Beds).

### Terms of Training.

Candidates for training at the Norfolk and Norwich Hospital must, except under special circumstances, be between the ages of 23 and 33. A good education is a necessary qualification, and it is considered desirable for probationers to be over 5 ft. 2 in. in height. If the first month's trial is satisfactorily completed, candidates are required to enter into an engagement to remain in the service of the hospital, to complete their training, for at least two years after their year of probation. An entrance fee of £10 10s. is required from all probationers, and no salary is paid during the first two years; for the third year £10 is given. The salaries of staff nurses range from £20 to £25, and of sisters, or head nurses, as they are more usually called, from £26 to £30. Lectures are given to nurses during their training by members of the hon. medical staff. Instruction in sick-room cookery also forms part of the curriculum. Nurses are required to present themselves for examination before receiving a certificate. Laundry and in as well as out-door uniform are provided by the hospital. By her agreement any nurse or probationer who breaks her engagement with the hospital, without due cause, is liable to a penalty of £3. Night duty is taken by the assistant nurses, *i.e.*, nurses in their second and third years, alternately two months' night and four months' day duty. No special probationers are received. For promotion to the charge of a ward a three years' certificate is essential. Sisters are selected for their general efficiency and administrative capacity.

### Hours On and Off Duty.

Probationers and assistant nurses go on duty in the wards at 7.30 a.m., and leave them in the evening at 9 p.m. They have two hours daily for recreation, from 2.30 to 4.30 or from 5 to 7, and time for meals has also to be taken from the above-mentioned hours. A whole day off once a month is allowed to nurses on day-duty, and those whose homes are near are allowed to go off on the evening of the day before, so that they may have a night away from the hospital. On Sundays nurses take their recreation time from 10 to 1, or from 2 to 5. Sisters' hours are the same except that on alternate Sundays they are off duty from 2 to 5 and from 2 to 10. Night nurses are on duty in the wards from 9 p.m. to 9 a.m., taking their time off from 10 to 12.30.

### Meals.

All meals are served in the nurses' dining-room, and no weekly "allowances" of food are given out to the nurses, Those nurses on night duty take stores for their midnight meal away from the dining-room each night after their supper. The hours for the day nurses' meals are as follows: Breakfast, 7; lunch, 9.45 to 10.45; dinner, 1.30 and 2 p.m.; tea, 4.30 and 5 p.m.; supper, 9 p.m. Night nurses have supper before going on duty at 8.45 p.m., and dinner at 9.30 a.m.

---

### Where to Go.

S. Cyprian's Home for Aged Women.—A sale of work, consisting of useful clothing and fancy articles will take place at the above home, 10, Little Park Street, Dorset Square, N.W., on Tuesday, Wednesday, and Thursday, November 22nd, 23rd, and 24th, between the hours of two and seven.

---

### Wants and Workers.

Mrs. A. Ward, Indian Industrial School, Battleford, Sask., N.W.T., Canada, asks if one of our readers will occasionally send her a copy of The Hospital? She says: "I miss the reading of The Hospital so much that I am writing to ask if you will allow me to ask your readers if one of them will kindly send me her paper, even occasionally. I know the postage would be rather more, but I am sure the sender would be repaid if she knew the pleasure the paper would give. In this far-off, isolated place any literature is welcomed—services of song, recitations, almanacs, pictures, games, periodicals, work patterns, &c., &c.—for in this "great lone land" it is impossible to go to shops to purchase these things. I used to send The Hospital occasionally to Canada. Now how glad I should be if someone would send it to me now and then. It is such a good paper. Good in every sense. Consumption, scrofula, bad eyes are very, very common amongst these Indians. Not many are strong and robust. Consumption seems a scourge everywhere."

### The East-end Mothers' Home,

394 AND 396, COMMERCIAL ROAD, E.

A USEFUL and well-managed charity is the East-end Mothers' Home. H.R.H. the Princess Frederica of Hanover is the patroness, and the Bishops of London and Stepney are president and vice-president, and the committee is an influential one. The design of the institution is to receive and nurse poor married women during childbirth, and to train midwives and monthly nurses for this work elsewhere. The resident staff consists of the lady superintendent, the house, district, and night sisters. The first has just completed her seventh, and the second her fifth year's work at the home. Five midwives and three monthly nurses are in training at a time, and go up for the quarterly examination of the London Obstetrical Society. In 1891 an out-patient department was added, and poor women are now nursed in their own homes for the sum of 3s. 6d. each; these small sums added to the £50 subscribed yearly by a lady for the purpose cover all expenses incurred by this department. In 1895 the back premises were rebuilt, and they now form a comfortable suite of receiving-rooms. Over them are situated bed-rooms for some of the nurses, and a tiny oratory, into which it is just possible to squeeze the members of the household for prayers. The nurses' rooms here are large and high; two are arranged for two nurses in each, and the third, curtained from the entrance, is devoted to one of the resident sisters.

When the patients are admitted they are taken into a cubicle, undressed, washed, and robed in dressing gown and slippers, and are then taken to their ward, whilst their own clothing is either stored in the cupboards built for this purpose, or, if very dirty, returned to their own people.

The home itself now consists of two houses with communicating doors between the wards. They are clean and bright with fresh paper, paint, and linoleum, but whilst every sanitary essential has been carried out under the honorary superintendence of Mr. Louis Parkes, nothing has been spent on adaptations or rebuilding. On each floor there are two large wards (the one containing four beds, the other three with room for an occasional one) and a small one for two beds. On the upper floor there is a nursery, where the babies' toilets are attended to, and a ward kitchen. Upstairs again is a sister's apartment—a cheerful room with all her personal treasures about—and the servants' sleeping quarters. On the ground floor are the lady superintendent's sitting-room, the sisters' sitting-room, and in the basement are the dining-room, kitchens, servants' room, &c. The home now accommodates eighteen patients, who are for the most part very grateful for the ministrations afforded them. They follow the fortunes of "our home," as they call it, with the keenest interest. A new paper on the wall, or a new piece of furniture, is an event, and old patients look forward to another admission with pleasure.

The duration of their visit is one fortnight. Home duties are so pressing upon the poor that the mothers cannot be absent longer than absolutely necessary from their families.

The home is supported entirely by voluntary contributions, the fees charged for training midwives and nurses being so small that it very little more than covers the cost of their maintenance. Additional furniture and household plenishings have been needed in consequence of the larger number of beds now available, and the Lady Superintendent has been fortunate in receiving many of the requisite articles for which she privately appealed to the members of her committee. If any amongst our readers happen to have any of the following articles to spare, they will be most acceptable and useful to the nurse and patients of this home. They are: Easy chairs (wicker, because these can be scrubbed), cane-bottom ward chairs, fireguards, bed linen. Money is also wanted for the purchase of additional surgical appliances (£5), six ward tables, and twelve wash lockers (£1 8s. 6d. each). Such contributions should be docketed for the furnishing fund, or they will be absorbed in the general accounts.

### Nurses and "Commissions."

THERE was a good custom affected by retail shopkeepers of the old-fashioned sort, according to which, when false coin was offered, it was not returned to the customer with a polite suggestion that it did not ring quite true, but at once, with a hammer and a mighty blow, was "nailed to the counter," as a warning to those who would attempt such tricks in future. Thus also should those be dealt with who attempt to tamper with a nurse's honour. The following letter was lately received by the matron of one of the largest of our London hospitals, the head of one of our largest training schools for nurses:—

"Dear Madam,—I write to tell you of my 'home,' and to ask the favour of your assistance. I have taken a house here at this address, with the intention of making it a nurses' co-operative institute and invalid home, &c. I could supply you with well-trained nurses from one guinea per week for all cases if you cared to send here for them. Of course the railway fare would make it a little expensive, but still I should think it might pay a London matron with a first-rate connection to have nurses from here and send them out from one and a half to two guineas per week. Do you think you could send me patients; and if so, would you take a percentage? This, of course, would be in strictest confidence. I would willingly give two shillings in the guinea for any convalescent or other patients sent me. I may add I am a trained nurse, so already have some connection.—I am, madam, yours truly."

Here we have a person posing as a "trained nurse," and so far mixing herself up with nurses as to have actually set up a "nursing institute," and yet knowing so little of the customs of a training school, or of the modes of thought or the standard of ethics to be met with among nurses, that she actually, as an ordinary matter of business, offers a commission or a tip (!) to the sacrosanct head of the whole establishment, and suggests that she should join with her in diddling the public! How can we wonder at the public sometimes speaking lightly of nurses when a person, posing as the matron of a nursing institute, can do such things? Let us nail such false coin to the counter at once.

### Minor Appointments.

VICTORIA HOSPITAL, KINGSTON.—Miss Adah C. Stronghill was appointed Matron of this hospital on the 10th inst. Miss A. C. Stronghill received her training at University College Hospital. She subsequently became charge nurse at Hampstead Infirmary, matron of Fleet Cottage Hospital, and is at present sister at the Royal Ophthalmic Hospital, Manchester.

RAINHILL COUNTY ASYLUM, NEAR LIVERPOOL.—Miss Daisy S. Y. Johnson was appointed Head Nurse and Matron of the above on the 11th inst. She was trained at the Bradford Royal Infirmary, where she obtained a medal for training. She was for some time theatre sister, and for nearly four years sister of female surgical wards of the same infirmary.

WESTERN GENERAL DISPENSARY, MARYLEBONE ROAD, N.W.—Miss C. Ethel J. Chaffer has been appointed Matron of this institution. She was trained at the Royal Infirmary, Manchester, and at Queen's Hospital, Birmingham. She has recently been charge nurse at the Park Hospital, Hither Green.

PRESTON AND COUNTY OF LANCASTER ROYAL INFIRMARY.—On November 7th Miss Clara Hoadley, who was trained at Guy's Hospital, was appointed Assistant-Matron of the above infirmary. She has been matron of Goole Hospital for two years.

ROYAL INFIRMARY, DERBY.—Miss H. Laing has been appointed Sister of out-patients at this institution. She was trained for three years at Liverpool Royal Infirmary.

### Lectures on Medical Relief.

UNDER THE AUSPICES OF THE CHARITY ORGANISATION SOCIETY.

### THE POOR-LAW INFIRMARY.

THE fourth lecture of this series was given at the Portman Rooms, on November 11th, by Dr. T. D. Savill, late Medical Superintendent of the Paddington Infirmary, the subject being "The Poor-Law Infirmary."

Dr. SAVILL prefaced his remarks by observing that it was one of England's proudest boasts that in the disposal of her social failures she was far in advance of other countries. The poorhouse was the first asylum for the sick and destitute; from that came the workhouse, and out of the workhouse were evolved the sick wards, and finally the separate infirmary for the sick. There were now twenty-four such separate infirmaries in London, with an aggregate of 12,332 beds, giving an accommodation greater than that of the general and special hospitals, which had together an aggregate of 10,883 beds. It was in 1864-5-6 that public attention was directed to the management of the workhouses through the efforts of Miss Louisa Twining, the Marchioness of Lothian, and the *Lancet*. Dr. Savill quoted from a report of Dr. Edward Smith, who, in attempting to show that things were not as bad as might be supposed, fully, if unconsciously, demonstrated their deplorable condition. In those days, before 1867, the medical officer lived away from the workhouse, and carried on a practice of his own outside, while the nurses were given to drunkenness and pilfering. Gathorne Hardy's Act, passed in 1867, established four important principles: First, that the sick should be cared for in separate buildings; secondly, that these separate infirmaries should be controlled by a medical man; thirdly, that the medical superintendent must reside on the premises and devote his whole time to the duties of his office; and, fourthly, that there should be an efficient, paid staff of properly-trained nurses with a properly-trained matron at their head.

With regard to the structure of the infirmaries, Dr. Savill took the Paddington Infirmary as an example of the class of buildings, and explained that they were erected on the pavilion system, in blocks, those for men and women being separated by the administration block, to which they were attached by bridges. Infirmaries had to provide for six classes of cases: (1) Severe cases, needing either special treatment or extra care in nursing, as with bed-ridden patients; (2) infectious or isolation cases; (3) children; (4) chronic cases; (5) lunatics (of which there were three classes, alleged lunatics, imbeciles, and idiots); and (6) lying-in cases. Speaking of the nursing staff, Dr. Savill gave the proper proportion of nurses relative to beds as that at Paddington, where, including the matron, the staff numbered 27 and the beds 284. He considered the present scheme of management of the infirmaries an ideal one when the officials were people of the right sort.

Admission into an infirmary in past days was an affair of difficulty and red tape, the signature of the relieving officer and of the district medical officer being necessary, even in cases of emergency, but this was now altered, and the medical superintendent had power to take in urgent cases on his own responsibility. The infirmary was a far less pauperising agent than the hospital, because of the strict inquiry system, which enabled payment to be insisted on in cases where payment was possible. Cases suitable for admission into an infirmary were, briefly, those not suitable for a hospital, such as chronic, incurable, infectious, and mental cases. As an ordinary rule operation cases should go into hospital. For actual experience of nursing, Dr. Savill considered that infirmary training offered better opportunities than that of a hospital, considering the number of helpless, bedridden patients, in the nursing of which the utmost care was needed to prevent bed-sores.

In considering the future, there were two points where improvement was required. In the first place, the demand for nurses was greater than the supply, to meet which difficulty the metropolitan infirmaries ought to train nurses for the provincial ones; and, secondly, there was great need for a further increase in the number of the medical staff; an increase in the residents, and, in Dr. Savill's opinion, the addition of a limited number of consultants. The latter step would be of immense advantage to the patients—how was it possible for three men to look after 600 beds?—to the ratepayers, for many cases might then be cured (and he gave an instance of a man who recovered after being bed-ridden for eleven years, costing the ratepayers during that time no less than £500), to the profession, and to the public. For teaching and research it would offer great opportunities. The student at the hospital sees for the most part special or selected cases, and leaves to take up his work knowing very little of how to treat a case of chronic bronchitis, or those ordinary cases which would form much of his every-day practice. In the infirmary wards he would see all kinds and sorts of diseases, and gain valuable experience in the study of neurology and mental disease, and those cases of neurasthenic insanity which never went beyond the workhouse. Dr. Savill strongly advocated that infirmaries should be thus thrown open to the medical profession, under regulation.

### The Book World for Women and Nurses.

[We invite Correspondence, Criticism, Enquiries, and Notes on Books likely to interest Women and Nurses. Address, Editor, THE HOSPITAL (Nurses' Book World), 28 & 29, Southampton Street, Strand, London, W.C.]

A DICTIONARY OF EMPLOYMENTS OPEN TO WOMEN. By Mrs. PHILLIPS and Others. (Published by the Women's Institute, 15, Grosvenor Crescent, S.W. Price 1s. 6d.)

THE public have always cause to be grateful to those who collect and afterwards reproduce useful knowledge in a handy form. Mrs. Phillips' little work, then, will surely be welcomed. It provides in a concise and uniform manner information respecting the various remunerative employments open to women. One of the chief deterrents which a would-be worker has to encounter is the difficulty of procuring information. It is necessary to know what qualifications are necessary, what conditions have to be accepted, and what is the remuneration and possibilities of advancement in any employment. It requires the knowledge which comes of much experience, instead of that of the youthful aspirant, to epitomise the pros and cons of any work, and enter it with the full knowledge of the conditions essential to contentment to the worker. Mrs. Phillips and her assistants have presented the result of their valuable experience and knowledge to women workers, who can now turn over the pages of the useful volume and learn the advantages and salaries offered, the training that is necessary, the hours of work, and where to apply for employment in any of the occupations dealt with. A perusal of the book will cause surprise at the numerous openings for women which now exist. They may also close it with pity when they have grasped how very hard it is for a woman without the shelter of a home behind her to earn a fair competency. The work closes with a list of training colleges and agencies.

### Everybody's Opinion.

[Correspondence on all subjects is invited, but we cannot in any way be responsible for the opinions expressed by our correspondents. No communication can be entertained if the name and address of the correspondent is not given, as a guarantee of good faith but not necessarily for publication, or unless one side of the paper only is written on.]

### GYNÆCOLOGICAL NURSING.

"A SUFFERER" writes: I have read with great interest the lecture on gynæcological nursing by Dr. A. Giles. I can speak from experience, having been a patient in the Chelsea Hospital twice within two years—last operation, hysterectomy was performed on me. The pain from the operation was nothing to the discomfort I experienced from having to wait for the use of the slipper and bed-pan, and several other patients in the ward complained of the same discomfort. The nurses were in no way to blame, they were most kind and patient under the circumstances, having only two slippers and one broken bed-pan amongst eighteen patients. I am quite sure it would give great joy both to nurses and patients if someone would see there were a few more slippers on the Albany Floor of the Chelsea Hospital.

"MATRON" writes: As matron of a hospital and a constant reader of your valuable paper, I am often struck by the way in which the medical profession desert their own subjects in lecturing, *i.e.*, anatomy, physiology, gynæcology, general surgery, and medicine, to encroach on the province of the trained matron by lecturing to probationers on nursing. It is needless to say that in consequence very ludicrous blunders have often to be recorded. For instance, in Dr. Giles' "Notes on Gynæcological Nursing," which appeared in THE HOSPITAL of October 29th, we see plainly that the writer is absolutely unaware that a slipper and a bed-pan are one and the same utensil! Again, he declares on the very insufficient credence of one person's assertion that it is a rule in hospitals to supply the bed-pans at certain fixed times only. Now the slightest inquiry would have shown Dr. Giles that although the bed-pans are taken round every four hours there never has been, nor is there ever likely to be, such a rule that a patient should not have the bed-pan at any other time. It is as little reasonable on the strength of one case to charge hospital nurses with causing unnecessary suffering to patients by their selfishness as it would be on the strength of one instance of a surgeon accidentally "snipping the bladder" in an operation, to charge the whole medical profession with entailing extra suffering by their clumsiness. Yet another point. If there is one thing more than another that it is essential to guard against in abdominal nursing, it is vomiting. Dr. Giles states that with a view to this end, the patient should be induced to take nothing by the mouth for at least twelve hours. But he subsequently suggests that the patient should, if much distressed by thirst, be allowed sips of hot water, "though it is not uncommon," he adds, "when the hot water has been taken in this way for it to be brought up ten or twelve hours after the operation." Contradictory directions such as these must prove very puzzling to the nurses. Dr. Giles further implies, though probably without intending to do so, that a patient once drank the contents of the hot water bottle at her feet, because she was left for a few minutes unwatched, at a moment when, according to him, "every want should be instantly supplied." But, seeing that, according to the same authority, this moment is also "a time at which people cannot be expected to act quite rationally," and when "some patients are apt to be hysterical," it is surely better that a judicious nurse should be guided at this juncture by her previous experience of abdominal cases, rather than by those "irrational" cravings whose gratification means injury to the sufferer.

### THE WORKHOUSE NURSE.

"AN INFIRMARY NURSE" writes: I would like to say a few words regarding the difficulty of obtaining nurses, and the unpleasant conditions under which they have to work. When the Local Government Board considered the matter and ordered the appointment of superintendent nurses, I thought that she, and she alone, was in any way subjected to the control of the matron. From the letter of "Workhouse Nurse," in THE HOSPITAL, November 5th, I conclude that such is not the case. The matter should be thoroughly reconsidered, for until the house and infirmary are entirely separate the question will always be a vexed one. Certainly all untrained matrons are not the ignorant, rude, and, objectionable women that "Workhouse Nurse" has happened to meet. Still, however capable an untrained matron may be in her own especial sphere, managing, supervising the able-bodied, dispensing stores, cutting out, &c., I myself would not be subjected to her rule in professional matters. The only way by which I think the difficulty could be met is by having a trained nurse as matron of the workhouse. Given full control of workhouse and infirmary a nurse should find sufficient to interest her and prevent her from getting into "a dull routine with warped energies." Why should the master and matron be compelled to be man and wife? Of course, if husband and wife can be got, and the wife a capable trained nurse, then the friction should cease, the nurse or nurses who were subordinate to the matron working together peaceably, for both would have nursing and all that pertained to it thoroughly at heart and try to further, and not retard its progress. When I propose this arrangement I am thinking only of small workhouses, which, I suppose, "Workhouse Nurse" also had in mind. Nurses in large unions, where the workhouse and infirmary are quite separate, have not such things to contend with. Fortunately, I have never worked under an untrained matron, but from nurses whom I have met, and one or two masters also, I feel sure that the grievances of the nurses are genuine, as is also the antipathy of the masters and untrained matrons for trained nurses. As regards complaints of patients against nurses, that is not a thing peculiar to patients who come into contact with untrained matrons, nor do I think that some of them would need much bribing. Everywhere are to be found some patients who will complain without a cause. Of course, officials should make it their business to know whether the inmates or patients are comfortable, but a great deal depends on the manner in which it is done. If a Guardian visits, speaks in an undertone to patients, asks them if they are comfortable and the nurses kind to them, any little grievance, real or imaginary, is at once exaggerated and the nurses' authority weakened. Everyone knows that the majority of trained nurses are drawn from a class who have been used to a moderate amount of comfort and the society and discipline of those with some education and refinement, and it must be most unpleasant to be now subjected to the control and supervision of the ignorant. Not that I wish to infer that "class" should count before capability. Certainly not! Still it is only right, and I think the duty of all Guardians to try to get people of high principles and education for the posts of authority. I sincerely sympathise with my colleagues working under disagreeable conditions, and should be glad to hear of someone approaching the Local Government Board on the matter.

### Appointments.

BIRMINGHAM AND MIDLAND EYE HOSPITAL.—We have received fuller particulars of Miss H. A. Marriott's appointment as Matron of the above. She was trained and certificated at St. Bartholomew's Hospital, afterwards serving a fourth year as staff nurse. She was sister of medical wards at the General Hospital, Birmingham, three years, and for the past twelve months has been assistant matron of that hospital.

LINCOLNSHIRE NURSING ASSOCIATION. — Miss B. M. Glover has been appointed Superintendent and Secretary of this association. She was trained at the Royal Infirmary, and at the District Home, Liverpool. She was afterwards Queen's nurse at Castor, and having trained for the L.O.S. certificate at the Victoria Home, Cheltenham, she became Queen's nurse, Huntingdon.

POOLE UNION WORKHOUSE.—We regret that in announcing Miss Jessie Hodges' appointment as Superintendent Nurse to the above, the name of her training school, *viz.*, the Royal Hospital, Sheffield, was incompletely given. Miss Hodges was sister at the Boston Regis Union Hospital.

JAMES MURRAY'S ROYAL ASYLUM, PERTH.—Miss Finch, matron of Dundee Royal Asylum, has been appointed Matron of the above institution.

### For Reading to the Sick.

"ONWARD and upward."

**Verses.**

Alas! long-suffering and most patient God,
Thou needst be surelier God to bear with us
Than even to have made us! Thou, aspire, aspire
From henceforth for me! Thou who hast Thyself
Endured this flesh-hood, knowing how as a soaked
And sucking vesture it can drag us down
And choke us in the melancholy Deep,
Sustain me, that with Thee, I walk these waves
Resisting! Breathe me upward, Thou in me
Aspiring! Who art the Way, the Truth, the Life—
That no Truth henceforth seem indifferent,
No way to Truth laborious, and no life,
Not even this life I live, intolerable!
—*E. B. Browning.*

Joy for the promise of our loftier homes!
Joy for the promise of another birth!
For oft oppressive unto pain becomes
The riddle of the earth. —*Burbidge.*

Thronging through the cloud-rift, whose are They—the faces
Faint revealed, yet sure divined, the famous ones of old?
"What?" they smile, "our names, our deeds—so soon erases
Time upon his tablet, where Life's glory lies enrolled?

Was it for mere fool's play, make-believe, and mumming,
So we battled it like men, not boy-like sulked and whined?
Each of us heard God's 'Come!' and each was coming,
Soldiers all, to forward-face, not sneaks to lag behind!

How of the field's fortune? That concerned our Leader!
Led, we struck our stroke, nor cared for doings left and right;
Each, as on his sole head—failer or succeeder—
Lay the blame or lit the praise: No care for cowards—Fight!"

Then the cloud-rift broadens, spanning earth that's under,
Wide our world displays its worth, man's strife, and strife's success;
All the good and beauty, wonder crowning wonder,
Till my heart and soul applaud Perfection—nothing less.
—*Browning.*

**Reading.**

"Seek that ye may excel."

An almost startling command; yet it is addressed to "all that in every place call upon the name of Jesus Christ our Lord," therefore unmistakably to ourselves.

Very likely our thoughts have been quite different from God's thoughts about it. We have been thinking it was useless to seek to excel, because we saw no likelihood of doing so; that it was presumptuous to think of such a thing; that it was even positively wrong to aim at it; yet, all the time, there, there the commandment stood, "Seek that ye may excel." For its right fulfilment there must be one preliminary and one object. The preliminary is that we must be "zealous of spiritual gifts." It is only when we are coveting earnestly the best gifts that the exercise and development of all others comes in its right place; that is, that we must be eagerly desiring and heartily striving and using His own means to grow in grace, to receive always more and more of His fulness, more light and love, more faith and power, more, above all, of His Spirit.

### Notes and Queries.

**The contents of the Editor's Letter-box have now reached such unwieldy proportions that it has become necessary to establish a hard and fast rule regarding Answers to Correspondents. In future, all questions requiring replies will continue to be answered in this column without any fee. If an answer is required by letter, a fee of half-a-crown must be enclosed with the note containing the enquiry. We are always pleased to help our numerous correspondents to the fullest extent, and we can trust them to sympathise in the overwhelming amount of writing which makes the new rules a necessity.**

**Every communication must be accompanied by the writer's name and address, otherwise it will receive no attention.**

*Invalid Resident.*

(84) I should be glad if you will kindly tell me how to get an invalid lady or gentleman, desirous of a comfortable Christian home, or nurses requiring a home or rest.—*A Regular Reader.*

Ask your doctor to recommend you, and advertise.

*Lessons in Massage.*

(85) Kindly give names and addresses where massage is taught.—*Sister Dora.*

See our advertisement columns, or write to the Secretary, Institute of Trained Masseuses, 12, Buckingham Street, Strand.

*A Queen's Nurse.*

(86) Kindly tell me if I am trained enough for the Queen's Jubilee Institution. I have had one year at a cottage hospital, one year at a workhouse hospital, two years' district nursing.—*S. A. U.*

The Queen Victoria Jubilee nurses are required to have one year's training in an approved hospital and six months' experience in district work. You are well enough trained, therefore, if your training school is approved. Write direct to the Secretary, Q.V.J.I.N., Queen Katherine's Hospital, Regent's Park, N.W.

*Appointment in Australasia.*

(87) Is there any home office where I could receive information about obtaining a hospital appointment in South Africa or Australia? I have been trained for three years and a half at the London Hospital.—*Laura D.*

There is no association for this purpose. Hospital appointments are frequently filled up by the matrons of the larger London hospitals, by secretaries of the nursing associations, or privately. The Secretary of the Colonial Nursing Association, the Imperial Institute, might give you information.

*Salisbury Treatment.*

(88) Could you kindly give me some information about the "Salisbury dietetic" treatment for invalids?—*Nurse Berrie.*

The so-called "Salisbury treatment" is a modification of the old Banting system—that is to say, it is essentially a "proteid," or meat diet. Its most useful application appears to be in cases of "rheumatoid arthritis," chronic rheumatism, neurasthenia or nervous debility, and in troubles of digestion. Speaking generally, the system is as follows: Three meals a day of meat prepared in this manner: About 12 ozs. of fresh meat (preferably rump steak) are minced finely, or passed through the sausage machine, and then cooked with plenty of nicely flavoured gravy for not more than 15 minutes, preferably in a copper stew-pan on the fire, or in a salt jar in the oven. The meat should be decidedly underdone. No fluid should be taken with the meal, and if the system is rigorously carried out nothing in the way of farinaceous food should be added. Generally, however, patients find it necessary to take one or two small pieces of dry toast. Halfway between meals at least a pint of hot water should be drunk, so that during the 24 hours the total should not be less than two quarts; this necessitates a pint the first thing in the morning and a pint the last thing at night.

*Offensive Feet.*

(89) I should be obliged if you would inform me if there is any and what cure for smelling feet?—*Subscriber.*

Wash the feet nightly with soap and water, and then bathe them, or dab them over with 1 in 20 carbolic lotion. Put on a pair of clean stockings every day, the feet of which have been well sprinkled with boracic acid, and let the boots be thoroughly dried every night. If the boots themselves are offensive, their insides may be rubbed with a rag damped with formalin solution or carbolic lotion.

*German Vapour Baths.*

(90) Would you kindly inform me how a German vapour bath is given?—*Anxious.*

There is no vapour bath especially "German." Give us a more accurate description of what you want.

*Massage.*

(91) Would you be so kind as to tell me if Dr. Stretch Dowse is still giving lessons in massage?—*G. M. E. B.*

Dr. Thomas Stretch Dowse's address is 14, Welbeck Street, Cavendish Square, W. He occasionally gives lessons to ladies in massage, when introduced by a medical man.

*Plague Duty.*

(92) Can you tell me how a nurse should apply for plague duty in India?—*I.*

The Revenue Secretary, The India Office, Whitehall.

*Short Maternity Training.*

(93) The Secretary of the British Lying-in Hospital, Endell Street, London, W.C., informs us that the period for training monthly nurses at that hospital is one month.

*Monthly Nursing.*

(94) S. H. must send name and address with query.

